# Pathophysiological and Therapeutic Association between Brain-Gut Axis and Irritable Bowel Syndrome: A Systematic Review

**DOI:** 10.12669/pjms.42.7.14944

**Published:** 2026-07

**Authors:** Usman Farooq, Ayesha Sadiqa, Shaista Arshad Jarral

**Affiliations:** 1 Usman Farooq, House Surgeon at Rashid Lateef Medical College, Lahore, Pakistan.; 2 Ayesha Sadiqa, Professor and Head of Physiology, CMH Lahore Medical College and Institute of Dentistry, Lahore. 311, CMH Lahore Medical College and Institute of Dentistry, Lahore, Pakistan; 3 Shaista Arshad Jarral, Professor and Head of Anatomy, CMH Lahore Medical College and Institute of Dentistry, Lahore. 311, CMH Lahore Medical College and Institute of Dentistry, Lahore, Pakistan

**Keywords:** Brain-gut axis, Dysbiosis, Enteric nervous system, FODMAP diet, Irritable bowel syndrome, Microbiota, Psychotherapy

## Abstract

**Objective:**

To identify the association among the gut, brain, and related microbiota, to reach the best-suited personalized management plan for Irritable Bowel Syndrome (IBS).

**Methodology:**

A systematic review was conducted by reviewing studies across multiple Databases, i.e., Scopus, MEDLINE, PubMed, Web of Science, ScienceDirect (Elsevier), Cochrane Library, Embase, and Google Scholar. The timeframe of selected publications was from 2007 to 2025. The results were extracted from 49 selected manuscripts using PRISMA guidelines. The review discussed the pertinent link between the brain-gut axis and IBS, in relation to etiology, clinical features, and underlying pathophysiological mechanisms, and an optimal management plan that aligns with the new concept of personalized health care, alongside evidence-based medicine.

**Results:**

IBS is a multidimensional ailment concerning gut hypersensitivity, hyper-immunity, imbalanced gut flora, and excessive anxiety or derailed psychology, each presented with a particular feature and associated with related etiology. Conventional therapeutic management benefits from reducing fermentation through a suitable diet plan, antibiotics to regulate gut flora, and neuroregulators that augment signaling pathways between visceral (gut-related) and central (brain) nervous systems. Stress-reducing interventions helped to decline the nociception and symptomatic-anxiety bursts. Upcoming advanced techniques such as Fecal microbiota transplantation (FMT), psychedelic-assisted therapy, traditional Chinese medicine, and the use of neuromodulator devices express possibilities to cure.

**Conclusion:**

IBS is a multisystem pathology triggered by gut dysbiosis, hyper-immune responses, visceral hypersensitivity, and stress-axis dysregulation. Thus, it is evident that a multimodal personalized management approach, including dietary, microbiome-targeted, pharmacological, and psychological therapies, is recommended for IBS, based on symptomology and etiology.

## INTRODUCTION

Irritable Bowel Syndrome (IBS) is an increasingly well-known gastrointestinal disorder that involves episodic pain in the abdomen linked with irregular bowel movements, and without any apparent pathology. Its prevalence is about 4-10% worldwide,^1^ with females being the predominant gender.^2^

Previously, IBS was considered a gut disorder without infection, presently, it has been recognized as a multifaceted condition involving abnormal functioning of the brain-gut axis (BGA), a complex network among central nervous system (CNS), gastrointestinal tract (GIT), and enteric nervous system (ENS).^3^ In fact, IBS patients can be classified according to what is known as the “seven cluster model”, based on pain, GIT symptoms, extraintestinal symptoms, and psychological comorbidities.^4^

The gut microbiota is a topic that frequently arises in brain-gut axis research. In most cases, IBS patients showed less diversity in their intestinal microbiome than healthy controls.^5^ However, reductions in *Bifidobacterium* and *Faecalibacterium*, genera known for their anti-inflammatory properties, accompanied by increases in *Enterobacteriaceae* and other potential pathogens, such as *Firmicutes*.^6^,^7^ Beyond composition, microbial toxins, e.g., short-chain fatty acids (SCFAs), low-pH hepatic secretions, and tryptophan-derived products, interact with gut function. A general decline in butyrate-producing microbes has also been found in IBS patients. 8 Variations in SCFA profiles have been linked to increased intestinal permeability, visceral hyperreactivity, and altered ENS functioning.^9^

The pathophysiology of IBS is due to visceral hypersensitivity i.e. gut’s hyperactivity in response to normal stimuli. Experimental studies with balloon distension techniques concluded that IBS patients report pain at lower thresholds compared to controls.^10^ The underpinning reason for this hypersensitivity is mainly dysregulation of signaling pathways in the ENS and afferent neurons, which further excite pain transmission to the brain (cingulate cortex, anterior insula, and amygdala), thereby causing exaggerated abdominal discomfort and enhanced nociception. This dysregulation of signaling pathways in the ENS suggested a comprehensive association between IBS and the brain-gut axis.^11^

Although the scale of the disease is vast and profound, because IBS places a considerable load on all wings of healthcare, i.e., financial burden, negative impact on overall health, leading to decay in patients’ life quality, and creating an adverse effect on society. The latest advanced therapies are designed to enable more holistic management through personalized health care based on etiology and disease symptomology. The review focuses on the pertinent association among the brain-gut axis, IBS pathophysiology, and its etiology. This will aid in developing an optimal management plan that aligns with the new concept of personalized health care and evidence-based medicine.

## METHODOLOGY

A thorough literature search was conducted between 2007 to 2025 using various search engines. PRISMA guidelines were followed to conduct a systematic review. The data were compiled from 56 included studies after careful screening. The study examines the pathophysiological relationship between etiological imbalances and the clinical characteristics of Irritable Bowel Syndrome (IBS) to analyze the most suitable management plan for patients with IBS based on their symptomology.

### Searched Databases:

For a comprehensive and detailed literature search, we carefully examined databases known for their content validity and relevance to gastroenterology, internal medicine, community medicine, general surgery, psychology, and psychiatry. The primary data search sources included PubMed, Scopus, Web of Science, Embase, Cochrane Library, Google Scholar, ScienceDirect (Elsevier), and MEDLINE. Every database provides exclusive journal collections and a variety of manuscript types, including mini-reviews, original studies, scoping reviews, experimental studies, case series, meta-analysis, case reports, opinions, and commentaries on the related topic, i.e., under search. The inclusion of linked studies was ensured by published information on etiology-specific mechanisms and newly emerging, more reliable, and suitable multifaceted treatment options related to the signs and symptoms of IBS.

### Time frame for selected Studies:

To target an optimal blend of fundamental and the latest advanced literature, the related articles were gathered through various databases, but the timeframe of all selected publications was from 2007 to 2025.

### Inclusion Criteria:

The included articles highlighted the signs and symptoms of IBS, along with associated disease pathology. Studies that used multi-dimensional management to address IBS. English was used as the language in all published articles. All regions or countries of the World were considered eligible for inclusion in the population. The included study types were mini-reviews, original studies, scoping or systematic reviews, experimental studies, case series, meta-analyses, and case reports on the IBS-brain-gut axis.

### Exclusion Criteria:

Studies that discussed IBS etiology in isolation (i.e., not consistent with the underlying disease mechanisms) or highlighted the old-school multi-array treatment options without describing their rationale for cure with respect to patient need assessment were rejected for inclusion. Editorials, opinions, and commentaries were also excluded. Studies with comorbidities were also excluded from the review.

### Studies included:

In the present systematic review, forty-nine manuscripts have been included.

### Data Extraction:

### Demographics of included study population:

Any patient, as a volunteer or study population, from any nationality and any age group who had been diagnosed with IBS was included. Females’ predilection was observed. The middle-aged group was more common, irrespective of ethnic or dietary upbringings.

### Etiological and Management Details:

To unfold the association among the gut, brain, and related microbiota, and to target the best-suited personalized management for IBS, each aspect of disease has been taken into account including, etiology, symptomology and the pathophysiological connective mechanisms. The causative gut microbiome, inducing inflammatory reactions, gut hyperactivity, and stress-related endocrine responses. Simultaneously, the pathological connection was explored in two ways, on one side with the featured sign and symptoms of IBS, and on the other side with the conventional and modern therapeutic options, where literature concluded the use of a multi-focused treatment protocol involving four primary dimensions: Dietary Interventions, Microbiome-Directed, as well as Pharmacological Therapies, and Psychological Therapies, obviously along with fulfilled the evidence based personalized health care needs established on clinical assessment. Those suggestive treatment approaches that have not yet been declared for use due to still-awaited RCT reports.

### Inferential Results and Handling of Bias:

To minimize bias, especially regarding preprint literature, unreviewed publications, and predatory journals, a manual search-and-refine technique was implemented. To avoid duplication count and publication bias, those manuscripts that did not meet the minimum standards for results and language after in-depth validation were excluded. Synthesis of results generated after careful data extraction. The GRADE technique (Grading of Recommendations Assessment, Development and Evaluation) was used to assess and ensure the trustworthiness and validity of the collected evidence.

## RESULTS

**Fig.1:**
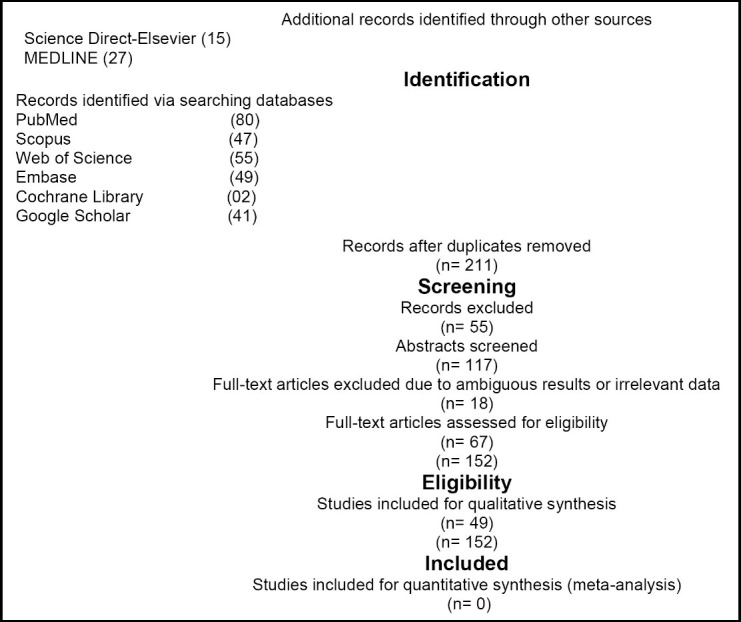
PRISMA flowchart for selected studies.

The current review was accomplished in accordance with the guidelines presented by Preferred Reporting Items for Systematic Reviews and Meta-Analyses (PRISMA) (Fig.1). Mainly, the literature was gathered obtained from latest peer-reviewed authentic publications.

The review discussed the pertinent link between the brain-gut axis and IBS, in relation to etiology, clinical features, and underlying pathophysiological mechanisms, and optimal management plan that aligns with the new concept of personalized health care, alongside evidence-based medicine. Evidence supports a personalized treatment approach combining dietary, microbiome, pharmacological, and psychological therapies. The review findings were gathered from the 49 included studies, with the central theme presented in Table-I.

**Table-I:** Presenting the pathophysiology of IBS, mediating the etiology with various management plans.

Etiological summary of IBS			
Causative Mechanisms of IBS	Key features	Pathophysiology	References
1. Gut Microbiota Dysbiosis	↓ Bifidobacterium / Faecalibacterium,	Increased intestinal permeabilityà visceral hyperreactivityàaltered ENS signaling	5-9
	↑ Enterobacteriaceae / Firmicutes		
	↓microbial diversity /SCFA imbalance		
2. Visceral Hypersensitivity	Heightened pain response to balloon distension	Abnormal signaling in ENS and sensory neurons → amplified CNS pain transmission	10, 11
	Lower perception thresholds		
3. Immune Activation	↑ Mast cells and lymphocytes near enteric nerves	Nociceptor activationàneuroimmune cross-communication and augmented local immune response	12-17
	Release of histamine, IL-6, TNF-α		
	Epithelial barrier dysfunction (‘leaky gut’)		
4. Stress and HPA Axis Dysregulation	↑ Cortisol and CRF	Alters gut motilityàsecretion, and sensitivityàstress triggers symptom flares	18-22
	↓ vagal tone		
	Heightened sympathetic activity
***Summarization of Management modalities of IBS***			
*Conventional Interventions*	*Therapeutic choices*	*Mechanism of Action*	*References*
1. Microbiome-Directed Therapies	Probiotics	Restores microbial balance + alters metabolite profilesàdecreases inflammation	23-27
	Prebiotics		
	Synbiotics		
	Postbiotics		
	Rifaximin		
	FMT		
2. Dietary Interventions	Low-FODMAP diet	Reduces fermentable substrates → ↓ gas, ↓ bloating; modulatesmicrobiota composition	28-33
	Soluble fiber (psyllium)		
	Elimination diets vitamin D		
	supplementation		
3. Pharmacological/ Neuromodulator Therapies	Antispasmodics	Modulate visceral sensitivityàgut motility improved, and pain signaling through ENS and CNS	34-41
	Peppermint oil		
	Eluxadoline (IBS-D)		
	Linaclotide		
	Lubiprostone (IBS-C)		
	TCAs		
	SSRIs		
	Serotonin receptor modulators		
4. Psychological Therapies	CBT	Reduces central pain perception and stress responseàrestores gut–brain communication	42-43
	Gut-directed hypnotherapy Mindfulness-based stress reduction		
	Relaxation training		
*B.Unique Integrative therapeutic approach*	*Research Support*	*Mechanism of Action*	*References*
1. Fecal Microbiota Transplantation (FMT)	Variable outcomes across trials with standardized donor selection and administration protocols	Rebalance gut microbial compositionàrestore diversity	44-45
2. Neuromodulation Techniques (TENS, sacral nerve stimulation, tDCS, rTMS)	Early pilot studies under investigation, as larger controlled studies needed to assess efficacy	Modulate visceral pain perception and autonomic balance	46
3. Traditional Chinese Medicine (WenTongGanPi Decoction)	Positive preclinical and early clinical data, Still multicenter RCTs required	Improve intestinal barrier integrity and reduce IBS-D symptoms	47
4. Psychedelic-Assisted Therapy (PAT)	Exploratory studies underway due to limited human data because of ethical regulations	Potential to reset maladaptive brain-gut network activity	48
5. Precision / Personalized Medicine	Conceptual and emerging, as integration into clinical workflow is still waiting for validation	Tailored therapy via microbiome, metabolomic, and genetic profiling	49

### Key Findings:

In most circles, IBS showed no links to inflammation or related processes, even though the older literature reported a link between gastroenteritis and the progression of IBS.^12^ Now, new evidence supports the immune activation, though not on a large scale; an excess of specific leukocytes, especially the lymphocytes and mast cells, has been found in the mucosa of the gut of IBS patients, mainly located near enteric nerve fibers, suggesting their role in neuro-immune communication.^13^ These cells release histamine, proteases, and cytokines (specifically IL-6 and TNF-α), which can activate nociceptors and trigger pain along with gut hypermotility.

Moreover, alteration of gut permeability, also called ‘leaky gut’, permits microbial products (lipopolysaccharide) to sensitize Toll-like receptors (TLRs), especially TLR4,^14^ finally augmenting the existing immune response in the gut mucosa.^15^ IBS patients expressed compromised epithelial and vascular barriers in their gut.^16^ These abnormal barrier mechanisms, activation of a localised immune response, and neuronal activation are increasingly recognised as key mechanisms responsible for brain-gut axis dysregulation in IBS.^17^

A key variable for brain-gut communication in IBS is the presence of psychosocial stress. In such patients, elevated levels of cortisol and corticotropin-releasing factor (CRF) indicate dysfunction of the hypothalamic-pituitary-adrenal (HPA) axis, which, further linked to hypermotility, hypersensitivity, and excessive secretion.^18^ Stress-induced overproduction of xanthine has been shown to trigger intestinal smooth muscle contractions, hence leading to symptoms typical of IBS-D.^19^ Certain aspects associated with stress in general, like anxiety, depression, and a history of adverse experiences of growing age,^20^ that not only cause but flare up the IBS symptoms.^21^ Imaging studies of the brain have validated these clinical observations, showing altered connectivity in brain regions that regulate stress and emotion, namely the amygdala, prefrontal cortex, and anterior cingulate cortex.^22^ These findings illustrate the complexity of the brain-gut axis, wherein psychological distress can trigger GIT symptoms.

The underlying mechanisms of IBS reflect an imbalance in the normal gut flora. Randomized controlled trials (RCTs) involving probiotics (*Bifidobacterium* and *Lactobacillus)* have shown alleviation of IBS presenting complaints, chiefly the bloating and the discomfort in abdomen.^23^ On the other hand, prebiotics, in particular galacto-oligosaccharides and fructo-oligosaccharides have been shown to promote proliferation of ‘good’ bacteria, also leading to gas overproduction.^24^ Recently, synbiotics (combinations of prebiotics and probiotics) and postbiotics (bacteria-derived metabolites) have been shown to reduce symptom severity.^25^

Antibiotic therapy, such as Rifaximin, has been evaluated in patients with diarrhea-predominant IBS (IBS-D) and has been shown to reduce bloating and loose stools.^26^ However, valid concerns exist regarding the longevity of the response and the risk of symptom recurrence. Another therapeutic intervention is fecal microbiota transplantation (FMT), though a negligible number of RCTs reported favorable clinical outcomes.^27^

Dietary modification is a cornerstone in IBS management, owing to the strong influence of ingested contents on brain-gut interactions. Among the studied interventions with low ‘fermentable oligosaccharides, disaccharides, monosaccharides and polyols’ (low-FODMAP) diet, which essentially cuts down on poorly absorbed carbohydrates that would usually accumulate in GIT, undergo fermentation, and generate gas. RCTs have shown that a low-FODMAP diet alleviates general IBS symptoms, particularly abdominal discomfort, bloating and improves stool consistency.^28^ However, concerns linger related to the longstanding feasibility of the nutritional plan and the possibility of loss of beneficial gut microbiota,^29^ thus execution of such nutritional plans would be advised under supervision.^30^

Additional dietary strategies like increased consumption of soluble fiber (psyllium), has been linked to the regularity of bowel movements, while the insoluble fiber may aggravate symptoms.^31^ Elimination diets driven by food intolerance testing e.g., gluten or lactose restriction can provide individualised benefit.^32^ Other novel dietary approaches may include; personalised nutrition plans guided by microbiome and use of vitamin D supplementation.^33^ Overall, dietary interventions remain first-line primarily because of their safety and cost-effectiveness.

## DISCUSSION

### Pharmacological treatment in IBS:

It is guided by the disease’s presentation (IBS-D, IBS-C, or IBS-M) and severity. Antispasmodics (hyoscine, mebeverine) are used to relief abdominal pain and cramping,^34^ similarly menthol is also renowned to encounter pain-related discomfort.^35^ For the IBS with diarrhea (IBS-D), loperamide (opioid receptor agonist), results in lowering stool frequency. Eluxadoline, is a similar drug, exhibited improvement in the consistency of stools and reduce pain in the abdomin.^36^ While for IBS with constipation (IBS-C), the use of linaclotide (guanylate cyclase-C agonist) and lubiprostone (Cl^-^-channel activator) caused regularity of bowel movements and relieved abdominal discomfort.^37^ In the same connection IBS-M represents IBS with mixed bowel response i.e. alternative episodes of diarrhea and constipation.^37^,^38^ for GLP-1 agonists have also shown potential in reducing pain.^38^

### Introducing New Management approaches:

Neuromodulation is crucial in IBS management; it actually unfolds the connection of brain-gut axis with actual perpetuation of clinical features. At low doses, tricyclic antidepressants (TCAs) can reduce visceral pain, and selective serotonin reuptake inhibitors (SSRIs) can improve both mood and bowel irragularities, particularly in IBS-C.^39^ Recent therapies using serotonin receptor modulators like tegaserod (5-HT4 agonist) and alosetron (5-HT3 antagonist) have been used in selected populations, but these drugs have safety concerns.^40^

Acupuncture techniques improved abdominal discomfort and stool frequency in selected patients with IBS-D.^41^ These medications often exert effects on mood, underscoring their role as neuromodulators.

Therapies for psychological interventions focus on dampening the central pain perception and the dysfunction of cognitive processes associated with GIT symptoms. Here, cognitive-behavioural therapy (CBT) is the intervention, broadly studied by the researchers, with proven efficacy in reducing abdominal pain, along with improving gut functions.^42^

Another proven technique, Mindfulness-based stress reduction (MBSR) and relaxation training, has shown potential in reducing stress-induced symptoms and improving existing coping mechanisms.^43^ To summarize, psychological interventions not only curtail IBS symptoms but also curb the high prevalence of associated comorbid anxiety and depression in this specific population.

### Study Limitations:

Fecal microbiota transplantation (FMT) remained a means of restoring microbial diversity and balance, though results of some trials still vary. This limits its clinical use due to the lack of standardized protocols.^44^ In the same connection, next-generation probiotics and synbiotics are under focused to replace the conventional probiotics in future.^45^

### Research For Future Directions:

Techniques aimed at neuromodulation, such as transcutaneous electrical nerve stimulation (TENS), sacral nerve stimulation, and, more recently, non-invasive brain current stimulation approaches (repetitive transcranial magnetic stimulation, transcranial direct current stimulation), are being assessed for their potential to modulate visceral pain perception and regulate autonomic balance.^46^ Apart from these, there is a rising interest in personalized and integrative medicine. A Traditional Chinese Medicine (TCM) approach known as ‘WenTongGanPi Decoction’ (WTGPD) has also shown some promise in alleviating IBS-D symptoms.^47^ A potential for Psychedelic-Assisted Therapy (PAT) as a possible intervention.^48^ Other approaches, including analysis of metabolites (aka metabolomics), microbiome profiling, and genetic risk markers, may enable tailoring therapy to individual patient phenotypes in the future.^49^ Collectively, these emerging therapies exemplify the shift from management focused solely on symptoms to a broader horizon of patient-centric management that targets the mechanisms that perpetuate disease manifestations.

## CONCLUSION

IBS is a disorder rooted in complex interactions between the gut, brain, and microbiome. Management has shifted from symptom-targeting to a multipronged approach that includes microbiota revision, diet, drugs, neuromodulation, and psychological care. These reflect a shift from GI-only to brain-gut communication issues. While current therapies help many, their limited efficacy underscores the need for more effective strategies. Emerging approaches such as fecal microbiota transplantation, probiotics, neuromodulation, and precision medicine show promise but require validation. Future management should be personalized to account for symptom variability and underlying mechanisms. Aligning innovative therapies with understanding causative processes can help achieve sustained relief and a better quality of life.

IBS is a multidimensional disorder that should also be treated with a multifaceted management approach. The prime concerns of gut hypersensitivity, hyper-immunity, imbalanced gut flora, and excessive anxiety or derailed psychology, based on related underlying etiology in IBS patients. A multifaceted treatment approach based on etiology may include reducing a suitable diet plan with rich focus on fermentation, antibiotics to regulate gut flora, and neuroregulators that augment signaling pathways between the gut and the brain. Alongside stress-reducing interventions helped to decline the nociception and symptomatic-anxiety bursts. Upcoming advanced techniques such as Fecal microbiota transplantation (FMT), psychedelic-assisted therapy, traditional Chinese medicine, and the use of neuromodulator devices express possibilities to cure.
